# The Temporal Relationship between Blood–Brain Barrier Integrity and Microglial Response following Neonatal Hypoxia Ischemia

**DOI:** 10.3390/cells13080660

**Published:** 2024-04-09

**Authors:** Arya Jithoo, Tayla R. Penny, Yen Pham, Amy E. Sutherland, Madeleine J. Smith, Maria Petraki, Michael C. Fahey, Graham Jenkin, Atul Malhotra, Suzanne L. Miller, Courtney A. McDonald

**Affiliations:** 1 The Ritchie Centre, Hudson Institute of Medical Research, Melbourne, VIC 3168, Australia; arya.jithoo@monash.edu (A.J.); tayla.penny@hudson.org.au (T.R.P.); yennie.pham@hudson.org.au (Y.P.); amy.sutherland@hudson.org.au (A.E.S.); madeleine.smith@monash.edu (M.J.S.); graham.jenkin@monash.edu (G.J.); atul.malhotra@monash.edu (A.M.); suzie.miller@monash.edu (S.L.M.); 2 Department of Obstetrics and Gynaecology, Monash University, Clayton, VIC 3168, Australia; 3 Department of Paediatrics, Monash University, Melbourne, VIC 3168, Australia; michael.fahey@monash.edu

**Keywords:** hypoxia–ischemia, blood–brain barrier, microglia, neuroinflammation, astrocytes

## Abstract

Blood–brain barrier (BBB) dysfunction and neuroinflammation are key mechanisms of brain injury. We performed a time-course study following neonatal hypoxia–ischemia (HI) to characterize these events. HI brain injury was induced in postnatal day 10 rats by single carotid artery ligation followed by hypoxia (8% oxygen, 90 min). At 6, 12, 24, and 72 h (h) post-HI, brains were collected to assess neuropathology and BBB dysfunction. A significant breakdown of the BBB was observed in the HI injury group compared to the sham group from 6 h in the cortex and hippocampus (*p* < 0.001), including a significant increase in albumin extravasation (*p* < 0.0033) and decrease in basal lamina integrity and tight-junction proteins. There was a decrease in resting microglia (*p* < 0.0001) transitioning to an intermediate state from as early as 6 h post-HI, with the intermediate microglia peaking at 12 h (*p* < 0.0001), which significantly correlated to the peak of microbleeds. Neonatal HI insult leads to significant brain injury over the first 72 h that is mediated by BBB disruption within 6 h and a transitioning state of the resident microglia. Key BBB events coincide with the appearance of the intermediate microglial state and this relationship warrants further research and may be a key target for therapeutic intervention.

## 1. Introduction

Perinatal brain injury caused by a severe hypoxic ischemic (HI) insult around the time of birth is a principal cause of altered brain development with long-term consequences for neurological health and function [1,2,3]. When the perinatal HI event is severe and/or prolonged, the subsequent neonatal clinical phenotype is termed hypoxic ischemic encephalopathy (HIE) [1,4]. Recent data from the United Kingdom show that the incidence of moderate/severe HIE is 2.07 per 1000 infants, reflective of the rate of HIE in high-resource countries and no change over time from earlier cohorts [5]. The sequence of pathophysiological events within the brain that leads to neonatal HIE is well characterized in preclinical and clinical studies, progressing through phases of primary energy failure, the latent period, and secondary and tertiary phases of injury [6,7]. Therapeutic hypothermia commenced in the latent period (within 6 h of birth) in term infants is the standard of care intervention for HIE, reducing the combined risk of death and disability significantly, but only partially [8]. Novel therapies must target alternate pathways that mediate the progression of neuropathology, potentially extending the window of opportunity for treatment. The neuroinflammatory cascade and neurovascular unit are strong candidates, given their regulatory contribution to neonatal HIE [9].

During the secondary phase of HI injury, the neuroinflammatory response is a principal mediator of progression of brain injury [2]. In response to HI, the brain’s microglia become activated, produce pro-inflammatory and anti-inflammatory cytokines, and release matrix metalloproteinases (MMPs), the latter of which lead to the breakdown of the blood–brain barrier (BBB) [2,3,4,5]. During the perinatal period, the immature BBB is highly vulnerable to injury, and the insult-mediated disruption of the BBB both contributes to and exacerbates neonatal brain injury [6]. As would be expected, the breakdown of the BBB is associated with cerebral hemorrhage and an influx of leukocytes, including neutrophils, lymphocytes, and monocytes [8]. The progression of BBB dysregulation is not well characterized in the neonatal brain following HI, but an understanding of the ontogeny of this process will allow for the tailoring of treatments that modify BBB disruption and repair.

The BBB exists as an integral component and the physical barrier of the neurovascular unit, which describes the physical interaction between the brain’s resident cells (neurons and glia) and the component cells of the vasculature [10]. The physical aspect of BBB function is characterized through brain micro-vessel endothelial cells interspersed with tight-junction protein complexes [10]. This barrier function prevents entry of red blood cells and peripheral immune/inflammatory cells into the brain. Proper functioning of the BBB is integral to maintaining a healthy brain environment, and BBB breakdown is associated with neuroinflammation and brain injury [11].

Characterizing the genesis of neonatal brain injury following an acute HI insult is central to being able to moderate injury progression and to test appropriately targeted therapies that are administered at an optimal time. Accordingly, in this study, we aimed to characterize the progression of injury to the BBB for 72 h following acute HI insult in the term-equivalent neonatal brain. To achieve this, we used the Rice–Vannucci model of neonatal HI injury in rat pups and assessed neuropathology at 6, 12, 24 and 72 h post-insult. We hypothesized that the structural integrity of the BBB would be critically impacted by HI injury, with distinct temporal changes in the cellular composition of the BBB and subsequent neuropathology. We assessed the somatosensory cortex region of the brain as this was the region where injury was primarily observed in this model of neonatal hypoxia ischemia.

## 2. Materials and Methods

Ethics approval:

All surgical and experimental procedures performed were approved by Monash Medical Centre Animal Ethics Committee A (MMCA/2019/28). All experiments were performed in accordance with the Australian National Health and Medical Research Council guidelines and informed by the international ARRIVE guidelines for the reporting of animal research.

Animals:

 Animal source and housing

Sprague Dawley rat pups were obtained from the Monash University Animal Research Platform (Clayton, Victoria) and were housed under standard conditions in the Monash Medical Centre Animal Facility throughout the experiment. Dams and pups were kept in a standard 12 h light/dark cycle with ad libitum access to food and water.

 Surgery protocol: modified Rice–Vannucci model to induce HI injury:

HI was induced in rat pups at postnatal day (PND) 10 using the Rice–Vannucci method of unilateral carotid artery ligation, as has been previously described [12]. As described in Table 1, there are 29 pups assigned to the sham group and 31 assigned to the HI injury group.

Briefly, pups were separated from their dam and placed on a 37 °C heating mat during perioperative and surgical procedures. The pups were initially anesthetized using 5% isoflurane in oxygen via inhalation for initiation and maintained at 2% for the duration of the surgery.

Each pup had a midline incision in the neck, and the left carotid artery was exteriorized and cauterized with an electrocautery device (Bovie Medical, Clearwater, FL, USA). The incision was closed using 6-0 polypropylene sutures (Covidien, Mansfield, MA, USA) and the isoflurane administration stopped. Bupivacaine with adrenaline (Aspen Pharmacare, St. Leonards, NSW, Australia) was applied to the suture site for pain management, and then a surgical glue was applied (VetBond, 3M, Sydney, NSW, Australia). Pups allocated to the sham group were anesthetized, and the artery was exteriorized without ligation. After surgery, all pups were returned to the dam for a 1 h recovery period. The pups that underwent ligation were placed in a humidified hypoxic chamber maintained at 35–36 °C for 90 min with an atmosphere of 8% oxygen in 92% nitrogen. The sham pups were kept on a 37 °C heating mat (body temperature was 35–36 °C) exposed to room air for the same duration as the hypoxic-treatment group. Following air or hypoxic treatment, the pups were reunited with the dam for recovery. Until the experimental endpoint, the pups were monitored and weighed daily.

 Post-mortem and brain processing

At each experimental endpoint (6, 12, 24, and 72 h), the pups were euthanized by an intraperitoneal injection of pentobarbitone sodium (Lethabarb, Virbac, Millperra, NSW, Australia). Brains were collected and immersion-fixed in 10% formalin for 48 h, processed, embedded in paraffin wax, and sectioned at 6 µm.

Immunohistochemistry

Specific concentrations and protocols for each antibody are listed in Table 2. Briefly, tissue was dewaxed using serial xylene and ethanol immersions, followed by antigen retrieval in a heated citric acid buffer (pH 6). Endogenous peroxidase activity was blocked by pre-incubating the tissue with hydrogen peroxidase to prevent non-specific background staining. Non-specific binding was further blocked by applying bovine serum albumin (BSA) or normal goat serum (NGS). Primary antibody was added to the tissue and incubated overnight at 4 °C.

The primary antibody was then washed off, and a secondary antibody was applied and incubated on the tissue. Streptavidin–horseradish peroxidase (Strep-HRP; Cytiva, Marlborough, MA, USA) was added to visualize the target of interest upon activation with 3,3-diaminobenzidene (DAB; MP Biomedicals, Santa Ana, CA, USA). Positive staining was characterized as brown staining on the tissue. Slides were coverslipped using DPX mounting media (Merck, Bayswater, VIC, Australia). Positive staining was characterized as brown staining on the tissue.

Gross brain morphology, hemisphere volume, and cerebral microbleeds were analyzed using hematoxylin and eosin (H&E) staining, neuronal cell counts were assessed using neuronal nuclear protein (NeuN), and microglia were identified using ionized calcium-binding adaptor molecule 1 (Iba1). Glucose receptors were identified using glucose transporter 1 (Glut-1), astrocytes were assessed using glial fibrillary acidic protein (GFAP), BBB integrity was measured by albumin extravasation into the brain parenchyma, and tight-junction integrity was assessed using claudin-5. Apoptosis was assessed using terminal deoxynucleotidyl transferase-medicated dUTP nick end labeling (TUNEL), and astrocyte–vessel interactions were assessed using a GFAP-laminin double label.

Immunohistological assessment:

For immunohistochemical analysis, brain sections were analyzed in duplicates, with three fields of view in the somatosensory cortex (approximately −3.3 mm and −4.7 mm from Bregma) analyzed per region. The data for each animal were averaged across duplicate slides and fields of view, and then individual animal datum was averaged across groups. Images were obtained using Aperio digital scanning (Leica Biosystems, Nussloch, Germany) at a 40× magnification by the Monash Histology Platform. All assessments were conducted on coded slides and images, with the examiner blinded to experimental groups.

Gross brain morphology, tissue area loss, and cerebral microbleeds were assessed with H&E (Trajan Scientific and Medical, Ringwood, VIC, Australia) stained sections. The volumes of the left (ipsilateral to the injury) and right (contralateral to the injury) hemispheres were measured using QuPath imaging software (University of Edinburgh, Scotland, UK, version 0.3.0). Percentage tissue loss was also calculated, measuring the difference in area between hemispheres using the following formula [12]:

*Area of the contralateral hemisphere − area of the ipsilateral hemisphere* *area of the contralateral hemisphere*

Cerebral microbleeds were quantified through a set of criteria used to maintain consistency between analyses and distinguish microbleeds from erythrocytes within vasculature that had been cut at a cross-section. Criterion was based on prior publications that qualitatively assessed microbleeds [13,14,15].

 
Microbleed criteria

*(a)*. Non-linear red blood cells.
*(b)*. Red blood cells surrounded by parenchyma.
*(c)*. Erythrocytes not contained within a vessel.
*(d)*. A total of >5 cells clustered together, <10 mm.
*(e)*. Exclusion of red blood cells from the choroid plexus and ventricles.

Albumin extravasation into the brain parenchyma was assessed for each brain region of interest; the total number of blood vessels with albumin extravasation was counted and the average for the entire region was obtained from duplicate sections. Neuronal integrity (NeuN+ cells) and cell death (TUNEL+ cells) were analyzed using an average for each animal between the fields of view and duplicate sections, presented as a mean for each group, displayed as cells/mm^2^. NeuN staining with a defined nuclear membrane and light cytoplasmic staining and morphology, that is, healthy neurons only, were counted [16].

Using manual counting, an analysis of the immunohistochemistry identified the number of positively stained cells within the somatosensory cortex using QuPath software. The quantification of microglia (Iba1+ cells) morphology states was separated into three distinct phenotypes [17]. Resting, ramified microglia were classified as Iba1+ microglia with the appearance of multiple complex and branching projections extruding from the cell body. Microglial cells in the intermediate process of activating were identified as a “bushy” phenotype, with a singular dendritic projection from the cell body. Fully activated cells were classified as ameboid, with a round distinct cell body with no dendritic projections [17,18].

Glucose transporters (Glut1+ staining), astrocytes (GFAP+), tight-junction markers (claudin-5), and basal lamina (laminin+) staining density in the somatosensory cortex were analyzed using QuPath and Image J software, as described previously [19,20]. Density was averaged between selected regions of interest per animal, and a mean was calculated for each animal.

Statistical analysis:

The results are expressed as the mean ± standard error of the mean. A statistical analysis was performed using GraphPad Prism 9.0 (GraphPad Software, San Diego, CA, USA). Experimental groups were checked for normality, and were compared using a two-way ANOVA, with age and injury as variables, and multiple comparisons were performed between row and column factors. Correlations between microbleed data at all time points against all major outcomes were performed using Pearson correlations, taking the R^2^ value and *p*-value. Data were deemed significant at *p* < 0.05.

## 3. Results

Animal Cohort Characteristics

A total of 60 rat pups (from 6 dams) were randomly assigned to experimental groups. To control for litter variation, littermates were divided between experimental groups. A total of 31 pups were exposed to HI insult and 29 pups underwent sham surgery with no ligation or hypoxia exposure. In experimental cohorts, sex was not balanced between groups as these animals were used over multiple studies; as such, this study was not powered to detect sex differences. All pups survived until post-mortem (Table 1).

Progression of neuropathology over time following perinatal HI.

**Figure 1:**
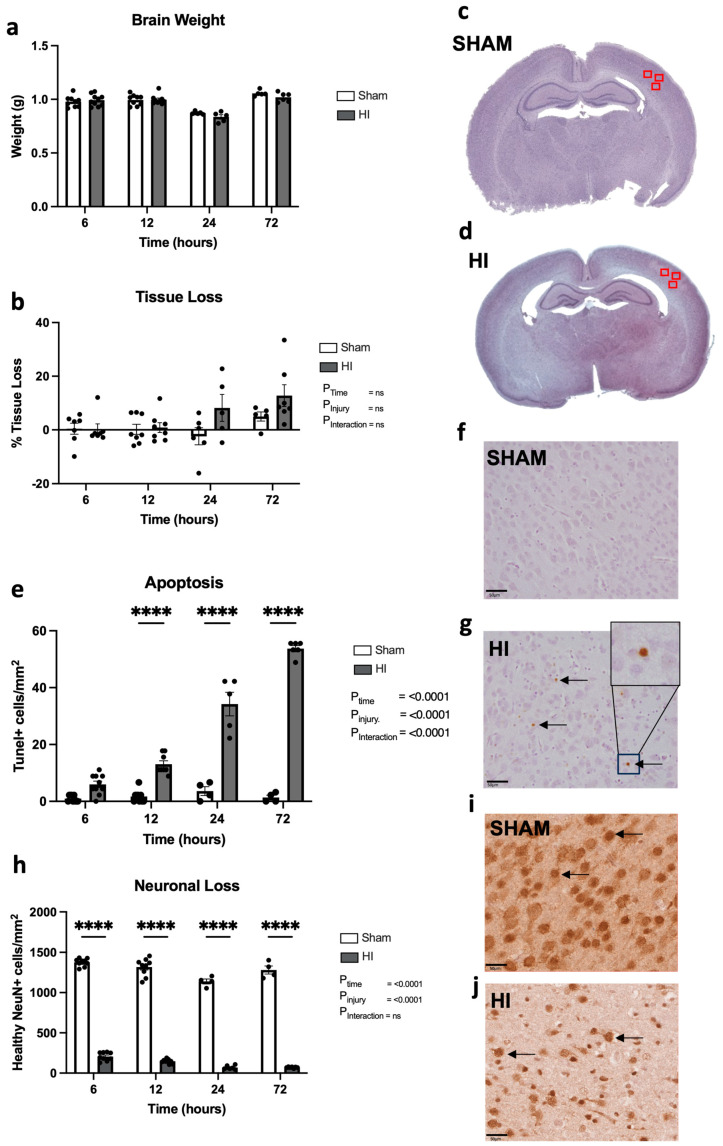
Morphological changes, temporal progression of cell death, and neuron loss from the brain following HI injury. Brain weight (**a**) and % tissue loss measured following hematoxylin and eosin staining (H&E) (**b**). Representative image of sham (**c**) and hypoxic–ischemic (HI) (**d**) brains used for tissue loss analysis, with regions of interest (somatosensory cortex (SSC)) depicted as red squares. Apoptotic cell death as determined by TUNEL-positive staining (**e**) in the SSC with representative images from the sham (**f**) and HI injury (**g**) groups at 72 h. Healthy neuronal cell count as seen by NeuN-positive staining (**h**) with representative images from the sham (**i**) and HI injury (**j**) groups at 72 h; black arrows denote apoptotic cells in (**h**) and healthy neurons in (**i**,**j**). **** *p* ≤ 0.0001, ns = not significant. Brain weight sham/HI—6 h n = 9/10, 12 h n = 9/9, 24 h n = 6/5, 72 h n = 5/7. Tissue loss sham/HI—6 h n = 7/7, 12 h n = 8/8, 24 h n = 6/5, 72 h n = 5/7. Apoptosis sham/HI—6 h n = 9/10, 12 h n = 9/8, 24 h n = 4/5, 72 h n = 4/6. Neuronal loss sham/HI—6 h n = 9/9, 12 h n = 9/9, 24 h n = 4/6, 72 h n = 4/7. Scale bar represents 50 µm. Data expressed as mean +/− SEM.

We firstly assessed gross brain morphology in response to acute HI, by measuring brain weight at post-mortem and tissue loss from the ipsilateral hemisphere on H&E-stained sections. There are no differences in brain weight between sham and HI injury animals at any time point (Figure 1a). Over time, there is a trend towards increasing left-hemisphere tissue loss in HI injury animals compared to the sham group (Figure 1b–d), but the response across animals is variable and this difference is not significant (*p* = 0.734 sham vs. HI at 72 h).

We assessed cell death following perinatal HI injury (TUNEL+ cells) in the cortex (Figure 1e–g). There is a significant increase in apoptosis over time (*p* < 0.0001), contributed to by a large increase in the HI injury group compared to the sham group (*p* < 0.0001), with a significant interaction between time and HI insult (*p* < 0.0001) (Figure 1e). At 12 h (*p* < 0.0001), 24 h (*p* < 0.0001), and 72 h (*p* < 0.0001), the HI injury group demonstrated significantly elevated cell deaths compared to the sham group; however, at 6 h, this increase was not significant (*p* = 0.06).

In combination with cortical cell death, we assessed the number of healthy NeuN+ neurons remaining in the cortex, identified as cells with a distinct cell body as well as a lack of nuclear fragmentation or pyknotic chromatin condensation [15] (Figure 1h–j). We found that there was a significant decrease in the number of healthy neurons in the HI injury group compared to the sham group (*p* < 0.0001), with a significant effect of time (*p* < 0.0001). A multiple comparisons test showed significant reductions in the HI injury groups compared to the sham group at 6 h (*p* < 0.0001), 12 h (*p* < 0.0001), 24 h (*p* < 0.0001), and 72 h (*p* < 0.0001).

Progression of microglial activation in the brain over time following perinatal HI

**Figure 2:**
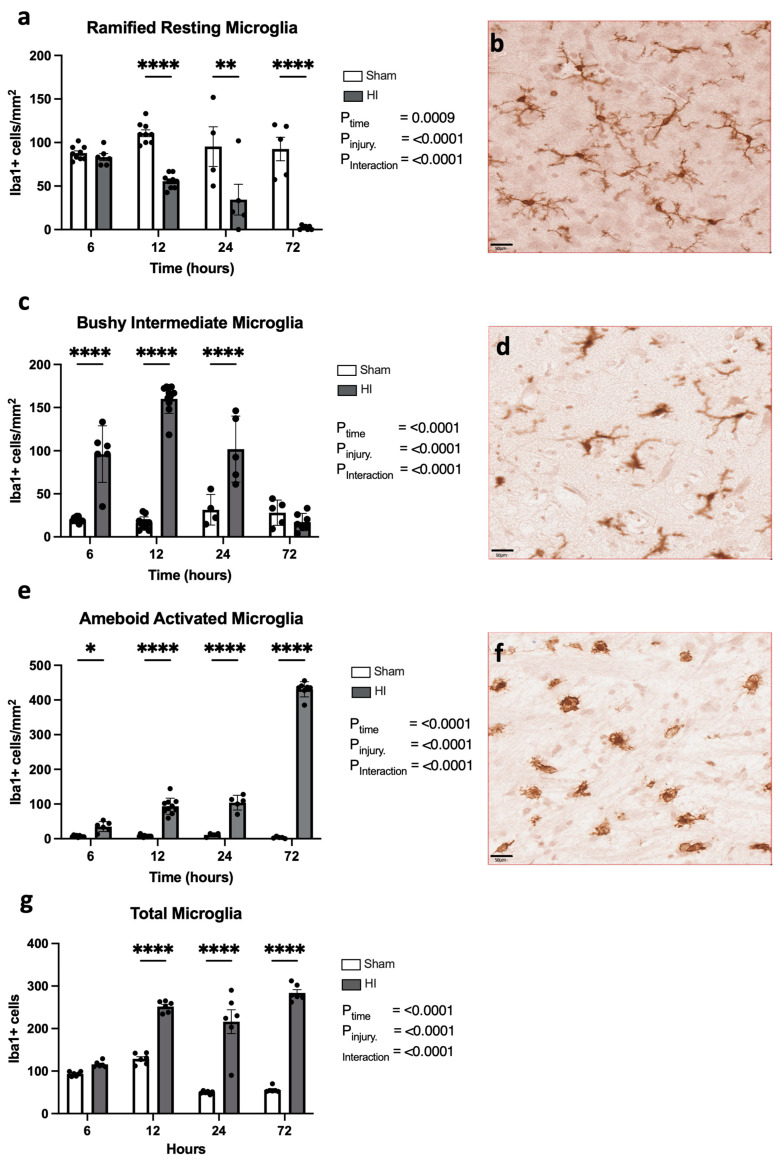
Temporal progression of microglial activation in the brain following perinatal brain injury. Number of Iba1+ ramified (**a**,**b**)-, bushy (**c**,**d**)-, and ameboid (**e**,**f**)-phenotype microglia in the somatosensory cortex (SSC) with respective representative images. Total microglial number in the SSC (**g**); * *p* < 0.05, ** *p* < 0.01, **** *p* < 0.0001, ns = not significant. Microglia sham/HI—6 h n = 9/6, 12 h n = 9/8, 24 h n = 4/5, 72 h n = 5/7. Scale bar represents 50 µm. Data expressed as mean +/− SEM.

Microglia were identified in the cortex with IBA1+ staining, where we categorized microglia according to phenotype—the resting “ramified” phenotype (Figure 2b), intermediate “bushy” phenotype (Figure 2d), and the activated “ameboid” phenotype (Figure 2f) are depicted in Figure 2.

The quantification of ramified resting microglia shows that there is a reduction in HI injury animals compared to sham animals (*p* = 0.0009; Figure 2a). There was also a significant effect of time (*p* < 0.0001), as well as a significant interaction between time and injury (*p* < 0.0001). Direct comparisons at each time point showed a significant reduction in resting microglia in the HI injury group compared to the sham group at 12 h (*p* < 0.0001), 24 h (*p* = 0.004), and 72 h (*p* < 0.0001); however, the 6 h time point was not different (*p* = 0.110).

The number of intermediate microglia that displayed a bushy phenotype was significantly increased in the HI injury animals compared to sham animals (*p* < 0.0001), and there was a significant effect of time (*p* < 0.0001) and significant interaction between time and injury (*p* < 0.0001). At 6 h (*p* < 0.0001), 12 h (*p* < 0.0001), and 24 h (*p* < 0.0001), there was a significant increase in intermediate microglia; however, by 72 h, there was no significant difference (*p* = 0.968).

Activated microglia, as evidenced by an ameboid phenotype (Figure 2e), demonstrate a significant effect of time (*p* < 0.0001), a significant effect of injury (*p* < 0.0001), as well as a significant interaction between time and injury (*p* < 0.0001). Tukey’s multiple comparisons test also showed significantly more ameboid microglia in HI injury groups compared to sham groups at 6 h (*p* = 0.0178), 12 h (*p* < 0.0001), 24 h (*p* < 0.0001), and 72 h (*p* < 0.0001). The results demonstrate a clear progression of the activation of microglial cells over the first 72 h following HI insult.

Total microglia cell counts were also quantified in the somatosensory cortex (Figure 2g). We found a significant overall effect of time using a two-way ANOVA, (*p* < 0.0001), a significant effect of injury (*p* < 0.0001), as well as a significant interaction between time and injury (*p* < 0.0001). Direct comparisons at each time point showed a significant increase in total microglial numbers in the HI injury group compared to the sham group at 12 h (*p* < 0.0001), 24 h (*p* = 0.004), and 72 h (*p* < 0.0001); however, the 6 h time point was not different (*p* = 0.8212).

Progression of HI-mediated BBB breakdown: Extravasation of peripheral blood

**Figure 3:**
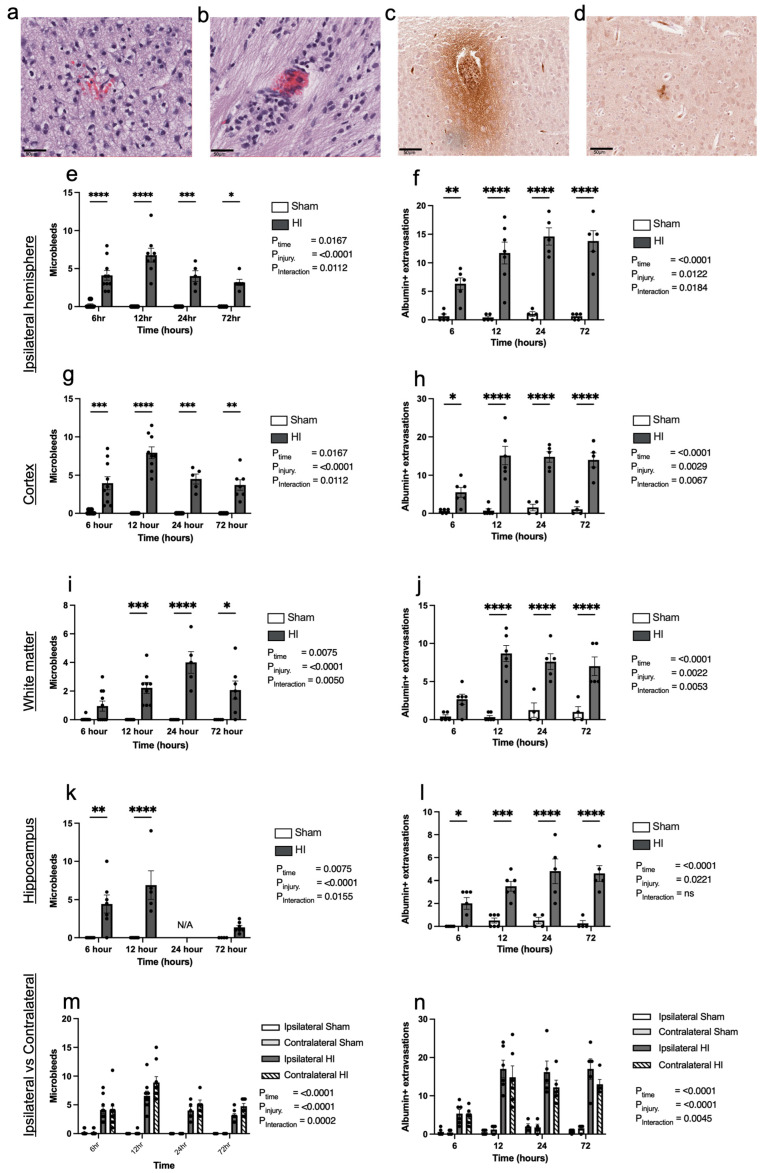
Extravasation of peripheral blood and albumin into parenchyma over time following perinatal brain injury. Representative images of extravasated red blood cells in the parenchyma (microbleeds), observed in the cortex (**a**) and white matter (**b**) at 72 h post-injury; scale bar = 50 µm. Representative images of albumin extravasations in the cortex (**c**); white matter (**d**) at 72 h post-injury; scale bar = 100 µm. (**e**) Microbleeds in the ipsilateral hemisphere. (**f**) Albumin extravasations in the ipsilateral hemisphere. (**g**) Microbleeds in the ipsilateral cortex. (**h**) Albumin extravasations in the ipsilateral cortex. (**i**) Microbleeds in the ipsilateral white matter. (**j**) Albumin extravasations in the ipsilateral white matter. (**k**) Microbleeds in the ipsilateral hippocampus. (**l**) Albumin extravasations in the ipsilateral hippocampus. (**m**) Comparison of number of ipsilateral and contralateral microbleeds over time. (**n**) Comparison of ipsilateral and contralateral albumin extravasations over time. * *p* < 0.05, ** *p* < 0.01, *** *p* < 0.001, **** *p* < 0.0001, ns = not significant. Microbleed sham/HI—6 h n = 9/10, 12 h n = 8/9, 24 h n = 6/5, 72 h n = 5/7. Albumin sham/HI—6 h n = 5/6, 12 h n = 5/7, 24 h n = 4/5, 72 h n = 5/5. Data expressed as mean +/− SEM.

To determine BBB integrity following HI brain injury, we examined brain sections stained with H&E for the presence of microbleeds and albumin extravasation, with representative examples of microbleeds shown in Figure 3a,b and albumin extravasation shown in Figure 3c,d. The analysis of the brain was segmented into structural areas: the entire ipsilateral hemisphere, and then a sub-analysis of the cortex, the hippocampus, and white matter.

Across the entire injured ipsilateral hemisphere, all HI injury cohorts demonstrate a significant increase in the presence of microbleeds and albumin extravasation (Figure 3e and Figure 3f, respectively) compared to the sham group. The two-way ANOVA results for microbleeds and albumin extravasation show a significant effect of time (*p* = 0.0167 and *p* = 0.0122, respectively), effect of HI insult (*p* < 0.0001 for both), and interaction between time and HI insult (*p* = 0.0112 and *p* = 0.0184, respectively). A post hoc analysis revealed significant increases in microbleeds and albumin extravasation in the HI injury group compared to the sham group at all time points: 6 h (*p* < 0.0001, *p* < 0.0033), 12 h (*p* = 0.0110, *p* < 0.0001), 24 h (*p* = 0.0007, *p* < 0.0001), and 72 h (*p* = 0.0178, *p* < 0.0001).

In the cortex, there is a significant effect of time on microbleeds (*p* = 0.0167; Figure 3g) and albumin extravasation (*p* = 0.0029; Figure 3h); there is a significant effect of HI injury (*p* < 0.0001 for both) and a significant interaction between time and injury (*p* = 0.0012 and *p* = 0.0067, respectively). There were significantly more microbleeds and albumin extravasation in the cortex at all time points in the HI injury cohort compared to the sham cohort at 6 h (*p* = 0.0002, *p* = 0.0195), 12 h (*p* < 0.0001, *p* < 0.0001), 24 h (*p* = 0.0011, *p* < 0.0001), and 72 h (*p* = 0.0201, *p* < 0.0001), respectively.

In the white matter, we observed a significant effect of time on the number of microbleeds (*p* = 0.0075; Figure 3i) and albumin extravasation (*p* = 0.0022; Figure 3j); there was a significant effect of HI insult (*p* < 0.0001 for both) and a significant interaction between time and injury (*p* = 0.0050 and *p* = 0.0053, respectively). There were significantly more microbleeds in the HI injury cohort compared to the sham cohort at 12 h (*p* = 0.0025), 24 h (*p* < 0.0001), and 72 h (*p* = 0.0100); however, there was no difference at 6 h (*p* = 0.6312). Similarly, there were significantly more albumin extravasations in the HI injury cohort compared to the sham cohort at 12 h (*p* < 0.0001), 24 h (*p* < 0.0001), and 72 h (*p* = 0.0001); however, there was also no difference at 6 h (*p* = 0.0553).

In the hippocampus, there is a significant effect of time on the number of microbleeds (*p* = 0.0075; Figure 3k) and albumin extravasation (*p* = 0.0221, Figure 3l), a significant effect of HI insult (*p* < 0.0001 for both), and a significant interaction between time and HI insult (*p* = 0.0155 and *p* < 0.0001, respectively). There were significantly more microbleeds in the HI injury cohort compared to the sham cohort at 6 h (*p* = 0.0029) and 12 h (*p* < 0.0001); however, no significant difference was identified at 72 h (*p* = 0.9933). The 24 h cohort was omitted due to a lack of viable tissue that contained hippocampi, as these brains had been used for a prior study. Albumin extravasation was significantly increased in the hippocampus in the HI injury cohort compared to the sham cohort at all time points: 6 h (*p* = 0.0104), 12 h (*p* < 0.0002), 24 h (*p* < 0.0001), and 72 h (*p* < 0.0001).

We also assessed the number of microbleeds and albumin extravasation present in the contralateral (non-injured) hemisphere and compared these to the injured ipsilateral hemisphere (Figure 3m and Figure 3n, respectively). Interestingly, microbleeds and albumin extravasation were also present in the contralateral hemisphere of the HI injury animals, and the number of microbleeds and albumin extravasation were comparable between the ipsilateral and contralateral sides. This phenomenon was not observed in the sham animals. While not significant, there did appear to be a peak in microbleeds at 12 h.

Progression of HI-mediated BBB breakdown: Structural integrity

**Figure 4:**
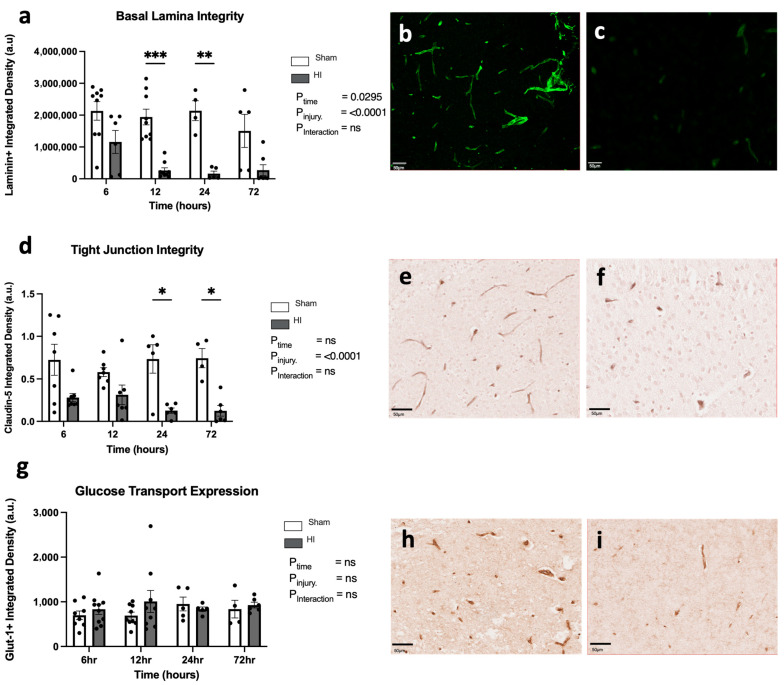
Breakdown of blood–brain barrier integrity. BBB basal lamina integrity identified by laminin-positive staining (**a**) with representative images from the sham (**b**) and HI injury (**c**) groups at 72 h; tight-junction integrity identified by claudin-5-positive staining (**d**) with representative images from the sham (**e**) and HI injury (**f**) groups at 72 h; membrane integrity identified through presence of glucose transporter Glut-1 (**g**) in the SSC with representative images from the sham (**h**) and HI injury (**i**) groups at 72 h. * *p* < 0.05, ** *p* < 0.01, *** *p* < 0.001, ns = not significant. Glut-1 sham/HI—6 h n = 8/10, 12 h n = 9/9, 24 h n = 5/5, 72 h n = 4/6. Claudin-5 sham/HI—6 h n = 7/8, 12 h n = 7/7, 24 h n = 5/6, 72 h n = 4/6. Laminin sham/HI—6 h n = 9/6, 12 h n = 9/9, 24 h n = 4/5, 72 h n = 5/7. Scale bar represents 50 µm. Data expressed as mean +/− SEM.

To assess the structural integrity of the BBB in response to HI insult, we measured multiple markers of BBB structure. The basal lamina of the BBB was identified with the use of laminin staining. The quantification of the basal lamina following HI injury shows a significant effect of time (*p* = 0.0295) and significant effect of HI injury (*p* < 0.0001), however a non-significant interaction between time and injury (*p* = 0.3287) (Figure 4a–c). Specifically, there was a significant decrease in laminin expression in HI injury cohorts when compared to the sham cohorts at 12 h (*p* < 0.0001) and 24 h (*p* = 0.0009) only.

Cellular tight junctions of the BBB were identified using claudin-5+ staining, with the analysis demonstrating a significantly decreased expression of claudin between sham and HI insult (*p* < 0.0001) groups, but no time effect (*p* = 0.4509) or interaction (*p* = 0.3176) (Figure 4d–f). There was a significantly decreased expression of claudin in HI injury groups compared to sham groups at 24 h (*p* = 0.0155) and 72 h (*p* = 0.0231) only.

Glucose transporters present in the endothelial membrane were identified using Glut-1+ staining. There is no significant difference observed in the expression of the glucose transporter glut-1 between sham and HI injury cohorts at any time point post-HI injury (Figure 4g–i), and there is no effect of time or injury.

**Figure 5:**
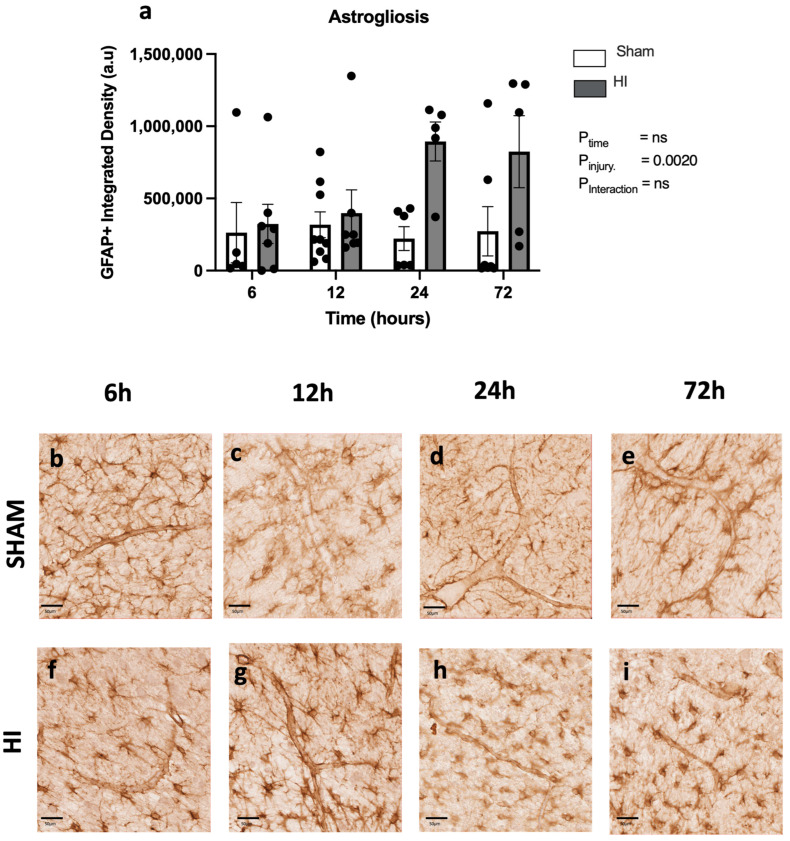
Temporal progression of astrogliosis following perinatal brain injury. GFAP density as a marker of astrogliosis in the somatosensory cortex (SSC) (**a**). Depiction of astrocyte end feet on vessels in sham groups (**b**–**e**) at each time point. Depiction of astrocyte end feet pulling away from blood vessels in HI injury groups at 24–72 h (**f**–**i**). Sham/HI—6 h n = 5/7, 12 h n = 9/7, 24 h n = 6/5, 72 h n = 7/5. Scale bar represents 50 µm, ns = not significant. Data expressed as mean +/− SEM.

GFAP-positive staining was used to determine astrocyte coverage in the cortex over the first 72 h following HI injury (Figure 5). We found a significant effect of HI injury using two-way ANOVA (*p* = 0.002; Figure 5a), with increased astrocyte coverage in the HI injury group compared to the sham group, but the post hoc analysis did not find any significant difference between sham and HI injury groups at any time point. Quantitatively, in the sham cohorts, close associations between astrocyte end feet and blood vessels can be observed (Figure 5b–e). Disassociations between the end feet and vessels can be observed at 24–72 h post-injury in HI injury cohorts, where astrocyte end feet are no longer connected to the vessel (Figure 5f–i).

Microbleed correlation analysis

To understand the mechanisms that likely contributed to the evolution of microbleeds in the first 72 h following HI brain injury, we performed Pearson correlations to identify which outcomes significantly correlated to microbleeds at each time point analyzed (Table 3). The strongest relationships with microbleeds (with R^2^ > 0.8) were intermediate microglia (R^2^ = 0.8465, *p* < 0.0001), activated microglia (R^2^ = 0.8247, *p* < 0.0001), and neuronal loss (R^2^ = 0.8546, *p* < 0.0001), all at 12 h. Other significant correlations (with lower R^2^ values) were identified and are documented in Table 3, including apoptosis, total microglia number, basal lamina integrity, tight-junction integrity, and albumin extravasation.

## 4. Discussion

**Figure 6:**
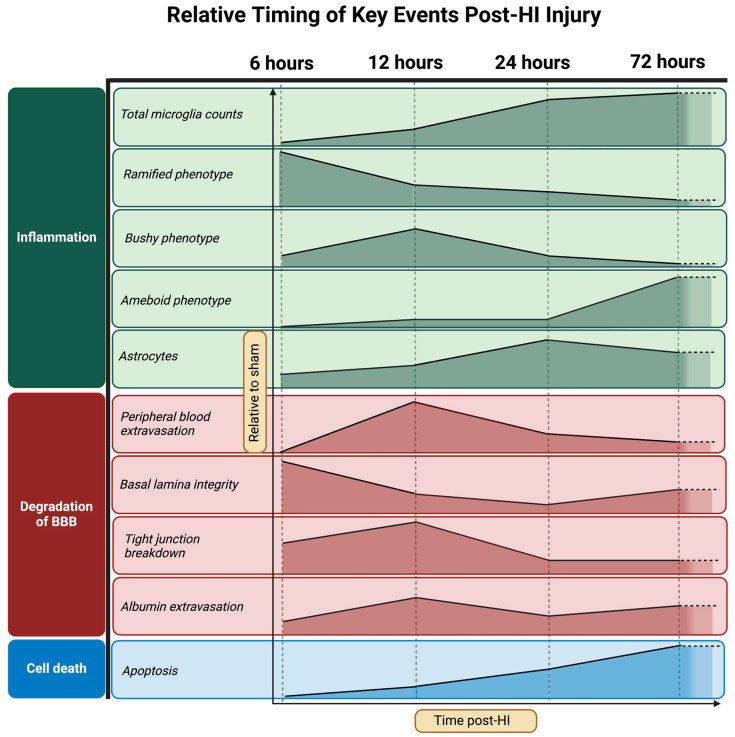
Relative timing of key events post-HI injury. Summary of the relative timing of key cerebral injurious events for the first 72 h post-HI injury, relative to sham.

The purpose of the current study was to examine the temporal response of the neonatal brain to an acute HI insult, with the aim of characterizing the timing and relationship between reduced BBB integrity, microglial cell activation, and neuronal cell health. The results demonstrate that, within 6 h of an acute HI insult, the integrity of the BBB is disturbed, allowing an influx of red blood cells into the cortical parenchyma with evidence of microbleeds and albumin extravasation, coupled with a significant shift in the phenotype of microglia towards an intermediate reactive cell type (Figure 6). Cellular apoptosis was not significantly increased at this early 6h time point, but the cell counts of healthy neurons were already profoundly decreased at this time, indicating that neuronal degeneration at this early time point is not mediated by apoptosis. At the later time points of 12, 24, and 72 h after neonatal HI, we show, for the first time, the evolution of neuroinflammation, with a progressive switch from a predominance of ramified microglia at 6 h post-HI injury toward an activated microglia phenotype at 72 h post-HI injury in cortical tissue.

Notable temporal changes were observed that are likely key mediators of both cerebral damage and repair; for example, at 72 h after neonatal HI, we observed the highest levels of cell death, the strongest presence of activated microglia, and the greatest reduction in tight-junction proteins in the BBB, as seen in Figure 4. When combined, the results of this study provide insights into the dynamic changes that contribute to neuropathology in the perinatal brain after mild/moderate HI insult, the likely mechanisms of neuronal degeneration, and indicate potential key targets for halting the progression of injury.

Despite the long-held view that the BBB of the immature fetal and neonatal brain is relatively leaky [21], it has now been established that the BBB of the term-equivalent neonatal brain is functional, providing a physical barrier against the unwanted entry of peripheral cells into the brain and actively mediating nutrient and other cellular exchanges across tight junctions [22]. While it is effective, it is the case that blood vessels in a young brain are more prone to injury than in the adult brain [22], thus allowing the leak of red blood cells and other products into the brain parenchyma. In the current study, we used the presence of red blood cell infiltration (microbleeds) and albumin into brain tissue as a primary assessment of the disruption of the BBB, together with an assessment of tight-junction proteins. We firstly noted that microbleeds and albumin extravasation were very rarely present in brains from sham animals. In contrast, a breakdown of the BBB was present in the ipsilateral hemisphere of HI injury animals, first evident at our earliest time point of 6 h post-injury. This timing concurs with the study of Muramatsu and colleagues who showed that the BBB remained relatively intact with protein extravasation at 3 h after HI injury in term-equivalent rat pups; but, by 6 h, there was protein leak into the brain, which continued to be elevated at 24 h [23].

We extended this finding and demonstrated that red blood cell extravasation peaks at 12 h post-HI injury in the cortex and hippocampus, and red blood cell extravasation remains significantly elevated at 24 and 72 h along with albumin extravasation. This elevated cortical and hippocampal disruption of barrier function is also seen in comparable literature that used albumin protein as a measure of BBB permeability [20,23,24,25], and we added to this by also showing that BBB breakdown was present in white matter, but with a delay in BBB disruption, which appeared to peak at around 24 h. A handful of previous ischemia studies have examined cerebral microbleeds in rodent studies using the visualization of albumin extravasation and fluorescein staining [26]; however, none specifically investigated RBC extravasation as a marker of microbleeds, and most have not examined the contralateral hemisphere and instead focus on the ipsilateral hemisphere [27,28,29,30]. In our model of mild/moderate neonatal HI brain injury, we found a significant presence of microbleeds in the hemisphere contralateral to the injury. We speculate that this may be a function of the model used, which subjects rat pups to whole body hypoxia. Furthermore, we have previously published that this model also induces systemic inflammation [12], which may further contribute to decreased BBB integrity in the “non-injured” contralateral hemisphere. While there may be a model or species effect, our findings of contralateral microbleeding correlate and are consistent with other rodent studies in the field [26].

As shown in Table 3, there is a high degree of correlation between the presence of microbleeds in the cortex, inflammatory cell profiles, and neuropathology, supporting the idea that the breakdown of the blood–brain barrier plays a central role in the progression of brain injury. The strengths of the associations between microbleeds and cellular apoptosis, neuronal loss, microglial activation, and albumin extravasation are the greatest between 6 to 12 h after insult, indicating that this is a critical window of opportunity for progression of injury, and for the potential of neuroprotective therapies. Secondary neuropathologies are upregulated or remain after 12 h, including a rise in ameboid microglia, astrogliosis and apoptosis, and the persistence of vascular tight-junction and basal lamina dysfunction; a schematic of this timeline is shown in Figure 6.

Structurally, the BBB comprises neurovascular unit cells, including vascular endothelial cells, the basement membrane, and tight junctions [28]. The basement membrane surrounds the endothelial cells and provides structural integrity [28]. Laminin is the non-collagen protein constituent of the basement membrane, and here we show that laminin density within the cortex reduces at 12 h after HI injury and remains suppressed at 24 h. A reduction in the density of the tight-junction protein claudin-5 was apparent from 24 h after HI. That laminin breakdown was preceded by reduced claudin-5 expression is not surprising, as other work has shown that the basement membrane does not regulate barrier function [31], but rather provides stability to other neurovascular unit (NVU) components, such as tight-junction proteins [32]. A further cellular component, glucose transporter Glut-1, facilitates the movement of glucose across the BBB, and seminal work has shown that Glut-1 is increased in response to acute hypoxia [33]. Our results do not show an overall change in Glut-1, albeit with a non-significant increase compared to the sham cohort at 12 h that does not persist, in keeping with an acute response [33]. Overall, we noted that red blood cell infiltration into cortical and hippocampal tissues was evident at 6 h post-HI, prior to significant changes in basement membrane laminin or tight-junction protein claudin-5. Ek and colleagues [24] examined three tight-junction proteins, claudin-5, occludin, and zona occludens-1 (ZO-1), at 6 h after neonatal HI, and showed that only occludin and ZO-1 were significantly reduced in the cortex. These tight-junction proteins provide the cellular connections between endothelial cells and, in doing so, occlude the extracellular space to create a barrier between cells [28]. The results of the current study are indicative of the complex responses and relationships of the cellular components of the blood brain barrier, providing novel insights into the timing of individual events.

As expected in the control animals, astrocytes are present around blood vessels—where close associations between the end feet and the vessel itself can be observed (Figure 5b–e). Our results show that, following HI, astrocyte end feet become disassociated from the blood vessels at 24–72 h post-HI. The clear dissociation of astrocytes from the vessels is indicative of a loss of contact between the end feet and the vessel post-injury (Figure 5f–i). This finding is consistent with Castillo-Melendez, Haruwaku, and colleagues, who showed similar findings [34,35] where they demonstrated astrocytic end-feet dissociation and impaired BBB function in conjunction with neuroinflammation. Similarly, the results presented in the current study add a critical timeline to these events and provide cellular points of interest for targeted interventions. BBB disruption is not solely a consequence of an injury, but rather also contributes to the perpetuation of the injury and is associated with a poor clinical prognosis [36].

Microglia, the resident immune cells of the brain, are key effectors in the regulation of BBB physiological function [37]. In their resting physiological state, they continuously survey the cerebral environment, which includes interacting directly with the endothelium of the BBB in order to maintain homeostasis through permeability changes [38]. In the ischemic state, local neuroinflammation prompts changes in the resident microglial profile as part of a rapid injury response and this inflammatory response is considered the most susceptible sensor of brain pathology [2], demonstrated by an alteration in morphological shape and the number of microglia [3,27]. The cytokines and chemokines produced by activated microglia after an ischemic event promote a neuroinflammatory state, which contributes to the impairment of the BBB through the degradation of its endothelial fenestrations [37]. In this ischemic state, perivascular microglia migrate towards the weakened BBB and contribute to the disintegration of the blood vessels. Microglia also interact with astrocytes and pericytes to further exacerbate neuroinflammation and BBB degradation [37,39,40]. We observed a temporal relationship in the microglia phenotype, where we firstly observed the quiescent, ramified state that transitioned into the typical ameboid activated state by 72 h post-insult. A notable observation was the swift, four-fold increase in the intermediate “bushy” phenotype of the microglia within 6 h after HI injury, which peaked at 12 h post-HI. Previous studies have shown 12 h post-HI injury to be a critical time point in the inflammatory response [41,42]. The bushy cells had a characteristic morphology of fewer processes extruding with indistinct cell body edges, giving cells a “bushy” appearance compared to the highly arborised, hyper-ramified state. Previous studies have described this characteristic morphology as being typical of an intermediate phenotype [17,43,44,45,46], and it has been suggested that they may have a functional inflammatory role [17]. However, this intermediate phase of the microglial phenotype is not well characterized, with the exception that this is a transient phase of activation and cell morphology [47]. In the current study, intermediate microglia were elevated between 6 to 24 h post-HI insult, while others observed similar intermediate bushy phenotypes at two days post-injury [46]. In our study, these intermediate microglia were no longer present by 72 h, indicating that their presence is indeed a transient phase in the injury process that may contribute to an escalation of neuroinflammation and subsequent neuropathology. Given their transient presence during key critical periods of injury progression, the specific therapeutic targeting of this intermediate microglial phenotype may be a very promising area of future research.

It has not been clear if the activation of microglia and associated inflammatory cascades play a direct role in inducing neuropathology, or whether cell activation is an observable response to the early changes associated with pathology [48]. In HI injury, the presence of ameboid microglia cells inducing cell death in immature white matter has been confirmed [49,50], and it has been postulated that they constitute the primary sources of proinflammatory cytokines in the brain [11]. In our study, we observed a large increase in the number of ameboid microglia at 72 h post-insult. By this stage, neuropathology was already evident, especially when taken in conjunction with other indications in our data, such as early neuronal death and the presence of microbleeds in the parenchyma. While not many experimental studies exist that sample microglial activation at early time points, our data proving a peak increase in activated microglia by 72 h post-injury are in line with the previous reports [51,52,53]. In addition to the transition between microglial states, we also observed a proliferation of total microglia, as evidenced by a 2.4-fold increase in total microglial cell counts within the cortex of HI-injured brains at the 72 h time point. The increasing proliferation of microglia over the time course of this study is in contrast with the peak of microbleeds in tissue, occurring at 12–24 h post-injury. It is possible that the reduction in microbleeds at 72 h post-injury might be attenuated by the increased presence of microglia in the tissue, as part of a red blood cell debris phagocytosis endeavor by the microglia, contributing to a decrease in microbleed presence over time [54].

We observed significantly reduced healthy neuronal counts as early as 6 h post-injury, suggesting that we saw end-stage cell death as an early response to HI injury. Furthermore, we also saw that gross cerebral pathology, such as hemispheric tissue loss, increased over time, with our data demonstrating an increasing profile of tissue loss over the first 72 h post-insult. In previous studies, we observed significant hemispheric tissue loss with the examination of neuropathology at 7 and 40 days post-injury [12,55], and thus, the earlier time points in the current study enhance the results to demonstrate that the earliest gross evidence of tissue loss occurs at ~72 h post-HI.

Current clinical treatment options for HI injury events are limited, and preclinical studies are required to investigate the cellular mechanisms of injury in order to develop effective therapeutics [56]. There are currently no delayed therapeutics available for neonatal HI injury. The only option available is therapeutic hypothermia, which has a narrow administration window to be effective—within 6 h of injury onset. Given our results, a clear target for future therapeutic investigations should focus on the 12–24 h period and potentially aim at modulating intermediate bushy microglia, as their emergence occurs around the key timing of events, such as the peak of microbleeds, the rapid reduction in healthy neurons, and the initiation of basal lamina breakdown. Typically, therapeutics focus on cell death, astrogliosis, and activated microglia, which we show to peak at 72 h. An understanding of the precise role of intermediate microglia and how their function can be modulated is essential and important for future therapeutic targeting. In addition, their role, if any, in the modulation of the neurovascular unit and BBB integrity should be considered.

## 5. Conclusions

We have shown the dynamic changes over the first 72 h post-HI injury of multiple indices of neuropathology. Neuronal injury occurs as early as 6 h post-HI insult, concomitant with the breakdown of the integrity of the BBB—with peripheral blood extravasating into the tissue in the ipsilateral hemisphere, cortex, and white matter. Microglia begin to transition to an intermediate phenotype in response to an injury, showing that neuroinflammation is upregulated by 6 h post-insult, with a peak increase in typical activated ameboid microglia by 72 h post-injury. We observed a change in the temporal profile of astrocyte attachment to the BBB. Markers of BBB integrity, such as the connection between cells in the basal lamina and their tight junctions, are shown to degrade significantly at 12–72 h post-injury. Based on our data, we speculate that microglial response is an early driver of the neuroinflammation that exacerbates BBB degeneration as a result of HI injury. We are constantly furthering our understanding of how neonatal brain injury progresses and how BBB dysfunction develops over time. We will be able to use these findings to best implement therapeutics that target these specific pathways—such as the emergence of intermediate microglia or micro-vessel breakdown—to improve the effectiveness of therapeutics that reduce neonatal brain injury.

## Figures and Tables

**Table 1:** Characteristics of the animal cohorts examined in this study, including number (n), sex ratio, brain weight at post-mortem, and pup mortality.

	6 h	12 h	24 h	72 h
Number (*n*)				
Sham	9	9	6	5
HI	10	9	5	7
Sex ratio (F/M)				
Sham	6/3	5/4	1/5	2/3
HI	5/5	6/3	1/4	4/3
Brain weight (g)				
Sham	0.97 ± 0.018	0.994 ± 0.016	0.87 ± 0.005	1.05 ± 0.012
HI	0.997 ± 0.015	1.006 ± 0.017	0.835 ± 0.021	1.018 ± 0.017
Pup mortality				
Sham	0	0	0	0
HI	0	0	0	0

**Table 2:** Experimental details for immunohistochemistry analysis.

Antibody	Target	Antigen Retrieval	Block Endogenous Peroxidases	Protein Block	Primary Antibody	Primary Antibody Concentration	Secondary Antibody
NeuN (Merck Millipore, Burlington, MA, USA; cat#MAB377)	Neurons	Heated in 0.1 M of citric acid buffer (pH 6) for 15 min and left to cool for 20 min	3% H202 in PBS for 10 min	5% NGS +1% BSA in PBS for 30 min	Mouse anti-NueN in 1% BSA + 0.3% TX-PBS	1:1000	Goat anti-mouse, biotinylated IgG
Iba-1 (Wako Pure Chemical Industries, Osaka, Japan; cat#019-19741)	Microglia	Heated in 0.01 M of citric buffer (pH 6) for 15 min and left to cool for 20 min	3% H202 in PBS for 30 min	10% NGS in 0.1 M PBS for 30 min	Rabbit anti-Iba-1 in 0.2% TX-PBS	1:1000	Goat anti-rabbit, biotinylated IgG
Glut-1 (Sigma-Aldrich, St. Louis, MI, USA; cat#ABI4683)	Glucose transporters	Heated in 0.01 M of citric buffer (pH 6) for 20 mis and left to cool for 20 min	0.3% H202 in PBS for 15 min	DAKO protein-free blocker for 1 h	Rabbit anti- Glut-1 in PBS	1:200	Goat anti-rabbit, biotinylated IgG
Glial fibrillary acidic protein (GFAP) (Sigma-Aldrich, St. Louis, MI, USA; cat#G3893)	Reactive astrocytes	Heated in 0.01 M of citric acid buffer (pH 6) for 9 min and left to cool for 15 min	0.3% H202 in 50% methanol for 20 min	5% NGS in PBS for 30 min	Mouse anti-GFAP in 0.3% in TX-PBS	1:400	Goat anti-mouse, biotinylated IgG
Laminin (Sigma-Aldrich, St. Louis, MI, USA; cat#NB300-144)	Basal lamina	1:500 proteinase K for 30 min at 37 degrees, humidified	N/A	10% NGS in 0.1% TX-PBS for 30 min	Rabbit anti-laminin in 0.1% PBST and 2% NGS	1:200	Goat anti-rabbit-488
Albumin (Waltham, MA, USA; A110-134A)	Albumin	N/A	3% hydrogen peroxide in 50% methanol for 10 min	5% NRS + 3% BSA in PBS-TX for 90 min	Sheep anti-rat albumin in 0.1% TX-PBS and 0.5% fish gelatin	1:2000	Rabbit anti-sheep

**Table 3:** All outcomes correlated to microbleeds in the cortex at all time points post-injury.

Outcome	R^2^ Value	*p*-Value	Confidence Interval
Tissue Loss			
6	0.003856	0.7948	−1.415, 1.099
12	0.008832	0.7107	−0.7536, 0.5258
**24**	**0.5758**	**0.0178**	**0.5229**, **3.969**
72	0.03043	0.5877	−2.060, 3.444
Apoptosis			
**6**	**0.6381**	**<0.0001**	**0.05811**, **0.1353**
**12**	**0.2987**	**0.0127**	**0.01686**, **0.1229**
**24**	**0.5821**	**0.0168**	**0.08507**, **0.6160**
**72**	**0.5488**	**0.0058**	**0.2593**, **1.177**
Neuronal Loss			
**6**	**0.4963**	**0.0008**	**−21.35**, **−6.823**
**12**	**0.8546**	**<0.0001**	**−13.09**, **−8.392**
**24**	**0.7372**	**0.0030**	**−18.51**, **−5.629**
**72**	**0.5729**	**0.0044**	**−27.99**, **−6.816**
Resting Microglia			
6	0.1253	0.1257	−0.3446, 0.04604
**12**	**0.7597**	**<0.0001**	**−0.6013**, **−0.3252**
24	0.3116	0.1183	−1.668, 0.2353
**72**	**0.5207**	**0.0081**	**−2.228**, **−0.4307**
Intermediate Microglia			
**6**	**0.4138**	**0.0022**	**0.4358**, **1.687**
**12**	**0.8465**	**<0.0001**	**1.030**, **1.630**
24	0.1907	0.2399	−0.4374, 1.477
72	0.1855	0.1622	−0.4843, 0.09319
Activated Microglia			
**6**	**0.3543**	**0.0056**	**0.2127**, **1.070**
**12**	**0.8247**	**<0.0001**	**0.5544**, **0.9131**
**24**	**0.5861**	**0.0162**	**0.2473**, **1.739**
**72**	**0.5959**	**0.0033**	**2.594**, **9.764**
Total Microglia			
**6**	**0.2622**	**0.0210**	**0.2590**, **2.799**
**12**	**0.7849**	**<0.0001**	**1.722**, **3.045**
24	0.1232	0.3543	−1.239, 3.029
**72**	**0.5158**	**0.0085**	**1.514**, **8.025**
Basal Lamina Integrity			
6	0.1603	0.0803	−1437, 89.98
**12**	**0.4751**	**0.0016**	**−2097**, **−596.6**
24	0.3064	0.1220	−1825, 268.3
**72**	**0.3470**	**0.0439**	**−2330**, **−39.61**
Tight-Junction Integrity			
6	0.07908	0.2297	−0.1054, 0.02702
**12**	**0.2830**	**0.0231**	**−0.05113**, **−0.004337**
**24**	**0.4918**	**0.0353**	**−0.1409**, **−0.006748**
**72**	**0.6159**	**0.0025**	**−0.1883**, **−0.05368**
Astrogliosis			
6	0.000642	0.9155	−97,873, 88,340
12	0.0173	0.6028	−36,306, 60,560
**24**	**0.511**	**0.0302**	**12**,**014**, **177**,**005**
72	0.01398	0.7143	−143,535, 201,924
Glucose Transport Expression			
6	0.01526	0.6039	−47.72, 79.76
12	0.03846	0.4354	−39.92, 88.32
24	0.01302	0.7701	−24.01, 31.09
72	0.01084	0.7474	−41.57, 30.81
Albumin Extravasation			
**6**	**0.5837**	**0.0006**	**0.5245**, **1.509**
**12**	**0.5975**	**0.0012**	**0.6644**, **2.082**
**24**	**0.4710**	**0.0412**	**0.07557**, **2.784**
**72**	**0.6678**	**0.0012**	**1.183**, **3.520**

## Data Availability

The dataset supporting the conclusions of this article is available upon request from the corresponding authors.
